# STROBE-GEMA: a STROBE extension for reporting of geographically explicit ecological momentary assessment studies

**DOI:** 10.1186/s13690-024-01310-8

**Published:** 2024-06-13

**Authors:** Célia Kingsbury, Marie Buzzi, Basile Chaix, Martina Kanning, Sadun Khezri, Behzad Kiani, Thomas R. Kirchner, Allison Maurel, Benoît Thierry, Yan Kestens

**Affiliations:** 1 École de santé publique, Université de Montréal (ESPUM), 7101 Av. du Parc, Montréal, H3N 1X9 Québec Canada; 2 Centre de recherche de santé publique (CReSP), 7101, Av. du Parc, Montréal, H3N 1X9 Québec Canada; 3 Université de Lorraine, INSERM, INSPIIRE, Nancy, F-54000 France; 4 Université de Sorbonne, INSERM, Institut Pierre Louis d’Epidémiologie et de Santé Publique IPLESP, Nemesis Team, Faculté de Médecine Saint-Antoine, 27 rue Chaligny, Paris, 75012 France; 5 Department of Social and Health Sciences in Sport Science, University of Konstanz, Universitätsstraße 10, 78464 Konstanz, Baden-Wuerttemberg Germany; 6 Centre for Clinical Research, Faculty of Medicine, The University of Queensland, Brisbane St Lucia, QLD 4072 Australia; 7 Department of Social and Behavioral Sciences, New York University School of Global Public Health, 726 Broadway, New York, NY 10012 USA; 8 Center for Urban Science and Progress, New York University Tandon School of Engineering, 6 MetroTech Center, Brooklyn, NY 11201 USA

**Keywords:** Geographically explicit momentary assessment, Guideline, GPS, Health, Environmental exposure, Review

## Abstract

**Context:**

While a growing body of research has been demonstrating how exposure to social and built environments relate to various health outcomes, specific pathways generally remain poorly understood. But recent technological advancements have enabled new study designs through continuous monitoring using mobile sensors and repeated questionnaires. Such geographically explicit momentary assessments (GEMA) make it possible to link momentary subjective states, behaviors, and physiological parameters to momentary environmental conditions, and can help uncover the pathways linking place to health. Despite its potential, there is currently no review of GEMA studies detailing how location data is used to measure environmental exposure, and how this in turn is linked to momentary outcomes of interest. Moreover, a lack of standard reporting of such studies hampers comparability and reproducibility.

**Aims:**

The objectives of this research were twofold: 1) conduct a systematic review of GEMA studies that link momentary measurement with environmental data obtained from geolocation data, and 2) develop a STROBE extension guideline for GEMA studies.

**Method:**

The review followed the Preferred Reporting Items for Systematic Reviews and Meta-Analyses (PRISMA) guidelines. Inclusion criteria consisted of a combination of repeated momentary measurements of a health state or behavior with GPS coordinate collection, and use of these location data to derive momentary environmental exposures. To develop the guideline, the variables extracted for the systematic review were compared to elements of the STROBE (Strengthening the Reporting of Observational Studies in Epidemiology) and CREMAS (CRedibility of Evidence from Multiple Analyses of the Same data) checklists, to provide a new guideline for GEMA studies. An international panel of experts participated in a consultation procedure to collectively develop the proposed checklist items.

**Results and developed tools:**

A total of 20 original GEMA studies were included in the review. Overall, several key pieces of information regarding the GEMA methods were either missing or reported heterogeneously. Our guideline provides a total of 27 categories (plus 4 subcategories), combining a total of 70 items. The 22 categories and 32 items from the original STROBE guideline have been integrated in our GEMA guideline. Eight categories and 6 items from the CREMAS guideline have been included to our guideline. We created one new category (namely “Consent”) and added 32 new items specific to GEMA studies.

**Conclusions and recommendations:**

This study offers a systematic review and a STROBE extension guideline for the reporting of GEMA studies. The latter will serve to standardize the reporting of GEMA studies, as well as facilitate the interpretation of results and their generalizability. In short, this work will help researchers and public health professionals to make the most of this method to advance our understanding of how environments influence health.

**Supplementary Information:**

The online version contains supplementary material available at 10.1186/s13690-024-01310-8.

**Table:** 

Text box 1. Contributions to literature
• We reviewed GEMA studies that link momentary subjective states, behaviors, and physiological parameters to localized environmental conditions
• Key pieces of information regarding the GEMA methods were either missing or reported heterogeneously
• This paper presents a STROBE extension guideline (STROBE-GEMA) to strengthen the reporting of GEMA studies

## Introduction

One of health promotion’s cornerstones is the belief that environments shape individual and population health [59]. While a strong body of literature indicates that various neighborhood characteristics such as greenness, walkability, food environments, presence of resources or neighborhood social dynamics contribute to population health and well-being [11, 20, 49, 57, 62], studies relying on fine-grained real observations of the health-place interaction remain scarce [4, 17, 46, 63].

The recent development and growing ubiquity of wearable technology has boosted our capacity to link measures of context to measures of subjective states, behaviors, and physiological parameters using *ecological momentary assessment* (EMA), also called *experience sampling method (ESM)* or, more generally, *ambulatory assessment* [7, 10, 52]. This method consists in collecting high-frequency data from participants in real-life settings using repeated short questionnaires that are most often answered on one’s mobile phone. The type of data collected is diverse, and may include people's thoughts or feelings, their momentary behavior, or self-reports about their social and environmental contexts [24]. EMA has been used to study a variety of outcomes, including physical activity patterns [48], smoking habits [47], eating habits [21], alcohol use [18] or psychological states [56], to name just a few. By design, EMA collects self-report data ‘at that moment’, hence improving ecological validity and eliminating recall bias. While such methods can help understand the processes underlying the generation of behavior and health outcomes, they can also provide key insights to develop ecological momentary interventions, that is, interventions that occur as people go through their daily lives [9, 15, 25, 51].

The growing prevalence and use of smartphones to administer EMA studies has opened up opportunities to collect additional sensor data. Today’s smartphones’ embedded sensors measure movement (accelerometry), light, noise, presence of other devices nearby (wifi, bluetooth) or location (GPS, Wi-Fi) [4]. Phone and app usage can also be tracked. Such contextual information can enrich our understanding of environmental correlates or triggers of health. Particularly, location data, once linked to environmental information through a geographical information system (GIS), allow researchers to measure local and momentary exposures. Because of their ability to assess context, studies that use such location information are called *Geographical or Geographically-explicit Ecological Momentary Assessments* (GEMA) [32].

GEMA studies are being published in various disciplines [28, 32, 35, 53, 65]. For example, GEMA helped explore the link between exposure to tobacco retail outlets and smoking urges. A closer proximity to tobacco retail outlets was associated with stronger smoking urge, but only when further than one mile away from home [58]. Similarly, more frequent exposure to tobacco retail outlets was linked to higher probability of lapsing among a sample of adults who had contacted a smoking quit line [30]. Another GEMA study conducted for a 48-h period on the Tangxia Street in the central area of Guangzhou, China, showed that exposure to noise was positively linked to momentary annoyance above 58 dB to 78 dB [66]. Clinical studies have also used GEMA. An example from psychopathology showed that the hallucination intensity among patients with schizophrenia and affective disorder was reduced when they were at work, but increased when they were involved in leisure activities [16].

GEMA studies present their own complexities. Beyond the temporal intricacies coming with repeated observations that characterize EMA protocols, GEMA studies further add the difficulty of collection and treatment of spatial data. Capturing spatial data implies issues about geographic accuracy and precision, or simply of missing data, due to variations in GPS receiver performance or environmental contexts [29]. Urban canyons - downtown streets with high buildings - are well-known for creating GPS inaccuracies, while being in a building or underground often means no GPS signal at all. Assisted-GPS — where cell tower or wifi location information further ‘help’ the device to define its location — can improve accuracy, but the diversity of smartphones and operation systems make accuracy assessments difficult [64]. Spatial data linkage for exposure assessment can also be done in many ways, raising issues of geographic ‘uncertainties’ [34, 50]. And finally, modeling GEMA outcomes also requires particular consideration, especially around issues of spatio-temporal resolution and dependence [12, 26].

Another consideration regarding the use of GPS tracking in such studies relates to ethical issues. Participants’ locations, trips, and more generally whereabouts are highly confidential, as these data could easily be used to identify individuals. Data security - transfer and storing - is certainly of utmost importance. Some ethical committees may impose some limitations on how the data is stored, for example asking to degrade spatial precision by blurring location precision. However, too much blurring might be an issue, depending on the research question. Therefore, these issues must be addressed systematically in GEMA studies. Fortunately, Bader et al. [1] and Fuller et al. [23] provide appropriate and inappropriate methods to collect online geographic data in the public health research field, such as data anonymization if research requires data linkage with identifying information.

Exploring the GEMA literature, we realized an important gap: there is currently no review of GEMA studies that reports on how GPS or similar location data is used to establish environmental exposure measures to be linked to outcomes in a momentary design. Furthermore, we found a lack of uniformity in the reporting of such studies. Often, key elements about either the study design, the data collection, the data linkage, or the temporal and spatial data analysis were seemingly missing or imprecise, limiting comparability and reproducibility. Recently, the CREMAS (CRedibility of Evidence from Multiple Analyses of the Same data) checklist was published, providing much needed instructions on how to report EMA studies [14, 36]. However, CREMAS does not address the spatial requirements that are core to GEMA studies.

Standardized reporting ensures transparency and reproducibility, allowing for the accurate interpretation and comparison of findings across different geographic contexts. Given the nuanced relationship between health outcomes and environmental factors, consistent reporting is essential for identifying patterns and trends that may have implications for public health interventions.

Moreover, clear reporting guidelines facilitate the integration of GEMA data into broader geographic information systems (GIS) and spatial analyses, enhancing our understanding of spatial-temporal dynamics in health-related behaviors and outcomes.

As a consequence of these gaps, this study has two objectives: First, to conduct a systematic review of GEMA studies that use a momentary design with sensor-based location data and make use of that data to establish measures of environmental exposure in relation to behavioral or health outcomes, and second, based on these review data, develop a STROBE-extension guideline for GEMA studies. We believe this work and guideline are crucial to improve not only the reporting quality of future GEMA studies, but also to help this field move forward.

## Methodology

The Strengthening the Reporting of Observational Studies in Epidemiology (STROBE) Statement gathers various guidelines for reporting studies using specific design. These reporting guidelines are described as “A checklist, flow diagram, or structured text to guide authors in reporting a specific type of research, developed using explicit methodology.” (https://www.equator-network.org/about-us/what-is-a-reporting-guideline/). Based on the recommendations of Moher et al. [43]’s *Guidance for Developers of Health Research Reporting Guidelines*, we employed essential strategies for developing our reporting guideline.

### Initial steps

#### Identify a need for a guideline

First, in our own work related to GEMA, we noticed that there was no official guideline regarding the reporting of GEMA studies. Because GEMA is a combination of EMA and GIS information, we believe it would be relevant to implement a more complete version of the CREMAS guideline, which has been designed only for EMA studies. A GEMA guideline would take into account the geographically-related elements of such studies. Since GEMA studies are quite new in the health research field, we conducted a systematic literature review of all of the studies using EMA and GIS. The idea was to identify key pieces of information that must be included in such a guideline, by finding geographically relevant elements, or the lack of details around them.

### Review the literature

#### Selection criteria

The systematic literature search adhered to the guidelines outlined in the Preferred Reporting Items for Systematic Reviews and Meta-Analyses (PRISMA). Scopus, PubMed and Web of Sciences were searched for all relevant GEMA studies ever published. The research equations were the following: Scopus: (("momentary assessment") AND ("location*" OR "GPS" OR "Global Positioning System*")); PubMed: ((Ecological Momentary Assessment[MeSH Terms]) OR ("momentary assessment"[Title/Abstract])) AND ((Geographic Information Systems[MeSH Terms]) OR ("Location*"[Title/Abstract] OR "GPS"[Title/Abstract] OR "Global Positioning System*"[Title/Abstract])); Web of Science: (("momentary assessment" OR "évaluation momentanée" (Title)) AND ("Location*" OR "GPS" OR "Global Positioning System*" OR "emplacement*" OR "Système de positionnement global" (Title)) AND (Article (Document Type)) OR ("momentary assessment" OR "évaluation momentanée" (Abstract)) AND ("Location*" OR "GPS" OR "Global Positioning System*" OR "emplacement*" OR "Système de positionnement global" (Abstract)). The search on PubMed and Web of Science was conducted on January 18th 2023, and Scopus on January 19th 2023.

#### Selection criteria

Studies were retained if they included**:** 1. Repeated momentary measures of variable(s) of interest along with momentary GPS coordinates, and 2. Momentary measures of environmental exposures calculated using the GPS coordinates. Variables of interest could be any outcome measured from short questionnaires sent to participants at the momentary level (e.g., depressive symptoms, cigarette craving, affective well-being, social interactions, pain, feeling of safety). Hence, evaluation of these outcomes needs participants’ active engagement in completing the questionnaire. Environmental measures could take various forms (e.g. measures of green space density, local social conditions, or distance to a feature of interest, to name just a few). Studies were excluded if outcomes were not captured through EMA/GEMA, or if studies had not reported any results about the momentary data. Papers with only descriptive statistics, reviews and protocol papers were excluded. We first screened the articles by reading all titles and abstracts, and when the abstract matched the corresponding inclusion criteria, full-text reads were done.

#### Data extraction

In order to organize the reported information in the included studies and to identify potential GEMA guideline items, the following variables of interest were extracted from each included article:

- *General study characteristics*: title, authors, journal, publication year, country of study sample, study rationale, target population, sample size, main outcome and main results
- *Key items from the Adapted STROBE Checklist for Reporting EMA Studies (CREMAS):* training, technology, wave duration, monitoring period, prompting design, prompt frequency, compliance, attrition, prompt delivery, latency, and missing data
- *Additional items related to EMA not included in the CREMAS checklist:* duration to complete prompt, number of items per prompt, momentary variables of interest, use of a validated measure instrument for EMA
- *Specific items related to geographic data collection:* proportion of response with GPS data, type of GPS data (momentary vs continuous), type of location data (GPS vs Wifi), technique used to derive momentary environmental exposure from location coordinates, type of environmental exposure

A coding form containing the variables of interest was developed and members from the core working team (MB, YK, SK, BK, CK, AM, GM, BT) extracted information from each study independently for different sets of items each. Ambiguities were discussed during weekly meetings within the group until a consensus was reached.

All variables extracted for the systematic review were compared with items from the STROBE and CREMAS checklists. Variables regarding relevant GIS information (specific to GEMA) which were not in STROBE were added to the different sections based on their placement in the included studies and discussions with the working group.

### Guideline first draft

The working team combined STROBE and CREMAS guideline items, and added items based on reporting GIS information in the literature review.

## Pre-consultation activities

### Identifying participants

In order to strengthen the validity and comprehensiveness of the tool, we searched for experts in the field of GEMA. Identification of experts was carried out using the list of authors from the articles included in our literature review. In addition to our seven member-working team, three experts participated in the elaboration of the guideline: one from Sport sciences in Germany, one from social et preventive health sciences in France, and one clinical psychologist and methodologist from the United States of America. Our own team is composed of geographers, health promoters, epidemiologists, and GIS experts. Efforts were made to have a multidisciplinary team, since GEMA methodology could be used in various health research fields.

## Consultation activities

### Conduct a Delphi exercise

Using an approach similar to a Delphi consensus, a Web-based survey was conducted. Presentation on topics underpinning the reporting guideline development was sent-out to experts. Then, experts were invited to separately rate the relevance, as well as the rationale for including the proposed items in the first draft of the guideline, based on our literature review. Experts were asked to comment about their views on the relative importance of the possible guideline items. Each experts’ comments were discussed by the core team which led to a revised version of the guideline. Each modification from the first version of the guideline was commented on, so the experts could understand the rationale of the proposed change. This second version was sent back to experts to be revised a second time. During the second round of the survey, each expert could comment on other experts' suggestions. If there was disagreement regarding specific items, a discussion was conducted until an agreement was reached among the main team members. Items were included if there was a consensus that the information was methodologically important to assess in a GEMA study, or if there was good evidence that it is frequently not reported. The order and the wording has also been assessed in the latest survey round. Thus, the development of the guideline required several iterations.

## Results

### Literature search

**Fig. 1:**
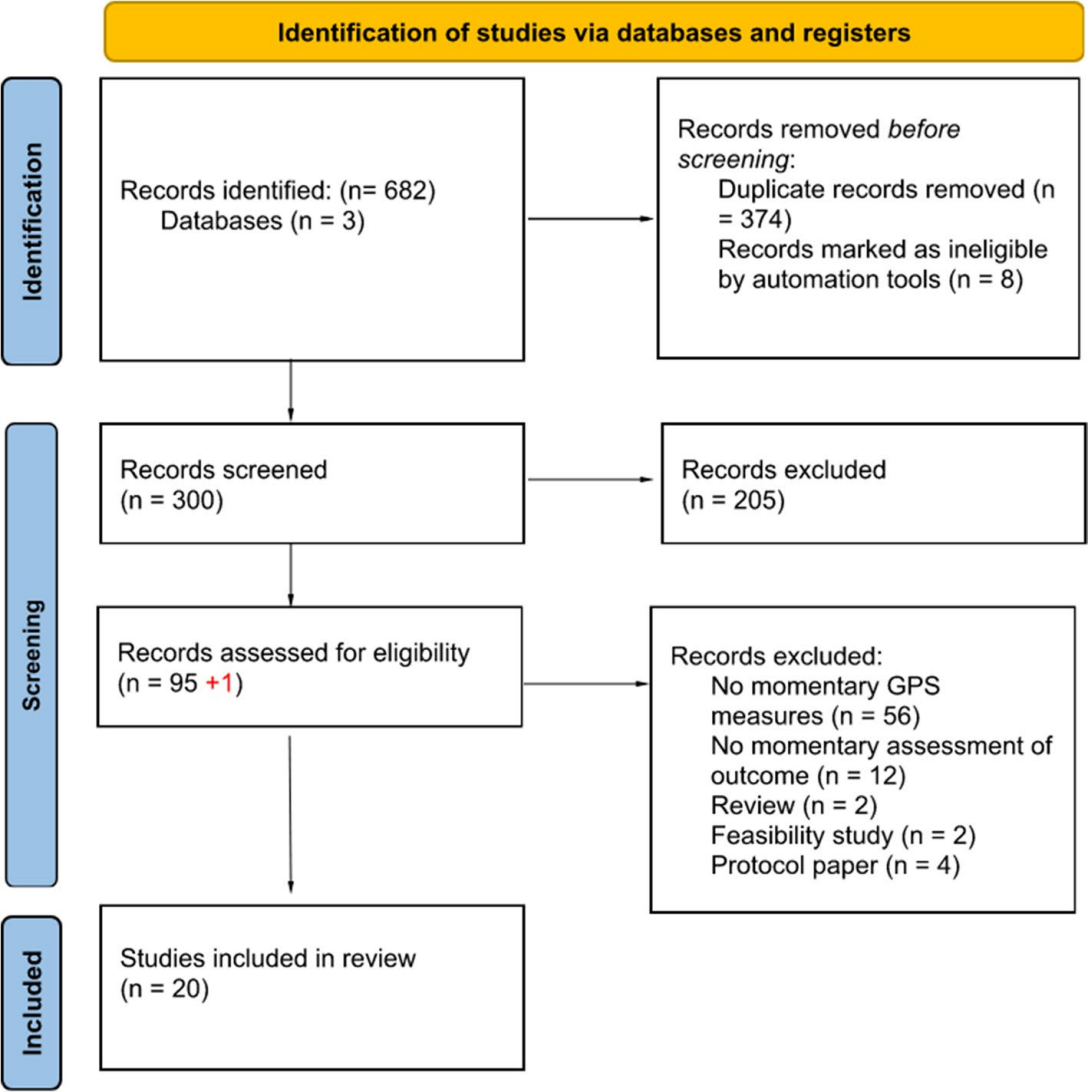
PRISMA flow diagram

A total of 308 potentially relevant original studies were identified throughout all three databases using our research equations, after the elimination of duplicate entries. Subsequently, we excluded 8 additional articles that were identified by our reference manager software as not being original studies, resulting in a total of 300 articles. We first screened the articles by reading all titles and abstracts, and when the abstract matched the corresponding inclusion criteria, full-text reads were done (a total of 96 articles). After careful evaluation of full-text, 20 articles met the criteria and were selected for inclusion in the review. Figure 1 provides a detailed flow chart of our screening steps.

Our results show important heterogeneity and inconsistency in the methodological reporting of GEMA studies, which could compromise reproducibility and comparison of studies’ quality. Among the reviewed studies, 4 (20%) did not report the technology used for data collection, and 9 (45%) did not mention whether devices belonged to participants or were provided by the study team. One study lacked key information regarding prompting design and only 4 (20%) studies specified the time window allowed to complete the survey. Most importantly, crucial information regarding GPS recording intervals was missing in 3 (15%) studies. Study schedule was fully described in all but two articles, which did not mention whether monitoring days occurred during weekdays and/or week-end days, and information on participants’ compliance was missing in 3 (15%) articles.

Moreover, we observed a diversity in prompting strategies, GPS recording intervals, and derivation of environmental exposures, which demonstrates the many ways in which the EMA methodology can be used to investigate associations between environmental exposure and individual experience.

Table 1 can be consulted for a summarized view of the literature review results. For more details, please see the Additional file 1, where complete information regarding elements from included articles are found. We will refer to included articles based on their numerated reference number in the following section.

**Table 1:** Design characteristics of the included GEMA studies

**ID**	**Study**	**Country**	**Target population**	**Sample size**	**Technology**	**Length of monitoring period (days)**	**Prompting design**	**Type of GPS data**	**Outcome**	**Type of environmental exposures**
1	Bollenbach et al [2]	Germany	Adults (general population)	49	Mobile phone	7	Event-based	Momentary	Affect	Outdoor environment
2	Byrnes et al [3]	USA	Adolescents	170	Application	12	Fixed Interval Contingent	Continuous	Substance use	Number of alcohol outlets
3	Clark et al [8]	USA	Adults (Clinical sample)	209	Application	30	Semirandom Interval Contingent	Not specified	Eating behaviour	Location type
4	de Vries et al [17]	The Netherlands	Adults (general population)	4318	Application	30	Event-based	Continuous	Happiness	Outdoor environment and weather
5	Li et al [35]	USA	Adolescents	155	Wearable device	4	Event-based	Continuous	Mood	Exposure to nature
6	Elliston et al [19]	Tasmania	Adults (general population)	72	Mobile phone	14	Random interval contingent	Momentary	Eating behaviour	Number of food outlets
7	Fulford et al [22]	USA	Adults (Clinical sample)	25	Mobile phone	7	Semirandom Interval Contingent	Continuous	Social interactions	Number of significant locations
8	Jacobson and Bhattacharya [27]	USA	Adults (Clinical sample)	46	Mobile phone	7	Fixed Interval Contingent	Continuous	Anxiety	Location type and weather
i	Kamalyan et al [28]	USA	Adults (Clinical sample)	88	Application	14	Semirandom Interval Contingent	Continuous	Happiness	Distance from home
10	Kondo et al [33]	Spain,United-Kingdom, the Netherlands, Lithuania	Adults (general population)	368	Mobile phone	7	Random interval contingent	Not specified	Mood	Natural outdoor environment
11	Mennis et al [40]	USA	Adolescents (Social-Spatial Adolescent Study)	139	Mobile phone	4	Random interval contingent	Momentary	Stress	Neighborhood disadvantage levels
12	Mennis et al [41]	USA	Adolescents (Social-Spatial Adolescent Study)	137	Mobile phone	4	Random interval contingent	Momentary	Substance use	Neighborhood disadvantage levels
13	Mitchell et al [42]	USA	Adults (Clinical sample)	10	Wearable device	7	Event-based	Continuous	Substance use	Distance from home and number of tobacco outlets
14	Mow et al [45]	USA	Adults (Clinical sample)	26	Mobile phone	60	Semirandom Interval Contingent	Continuous	Substance use	Time spent at home
15	Palmer et al [46]	Worldwide	Not reported	270	Mobile phone	35	Not reported	Continuous	Well-being	Activity space and spatial segregation
16	Su et al [53]	China	Adults (general population)	144	Mobile phone	2	Fixed Interval Contingent	Momentary	Happiness	Population density, points of interest density, and exposure to greenness
17	Tao et al [54]	China	Adults (*Meiheyuan* Community)	117	Mobile phone	12	Fixed Interval Contingent	Continuous	Stress	Location type
18	Tao et al [55]	China	Adults (*Meiheyuan* Community)	117	Mobile phone	2	Fixed Interval Contingent	Not specified	Stress	Outdoor environment
19	Watkins et al [58]	USA	Adults (Clinical sample)	47	Mobile phone	7	Random interval contingent	Momentary	Substance use	Number of tobacco outlets
20	York Cornwell and Goldman [61]	USA	Older adults	61	Mobile phone	4	Semirandom Interval Contingent	Continuous	Pain and fatigue	Neighborhood disadvantage levels

### General characteristics of the studies

The studies encompassed a wide range of topics across disciplines. Several studies explored the associations between environmental factors and various aspects of human experiences, including social interactions (*1, 3, 7, 9, 11*) and exposure to natural environments (*1, 4, 5, 10, 18*). Some studies focused on specific populations, such as youth (*2, 5, 11, 12*), older adults (9), or clinical populations such as individuals with schizophrenia (*7, 8, 14*). The 20 studies were published in 20 different journals, underlining the wide applicability of GEMA across research topics and disciplines, with significant growth in recent years: while the earliest article was published in 2013, 13 were published between 2020 and 2022.

### Geography of studies

Eleven studies were conducted in the United States (*2, 3, 5, 7, 8, 9, 12, 13, 14, 19*), three in China (*16, 17, 18*), one in four European countries (Spain, the Netherlands, the United Kingdom and Lithuania) (*10*), one in Germany (*1*), one in Tasmania (*6*), and one in the Netherlands (*4*). Additionally, one pilot study was conducted worldwide, including participants from thirteen different countries (*15*).

### Target populations

Several studies recruited participants within specific age ranges: 13-14 years old (*12*), 14-16 years old (*2*), above 18 (*1, 4, 10, 16*), between 18 and 60 (*17, 18*) or above 50 (*9*). Some studies also targeted participants with specific characteristics, such as socioeconomically disadvantaged adult smokers willing to quit (*19*), adults without underlying physical or mental health conditions (*1*), adults with no history of eating disorders or dieting (*6*) adults diagnosed with attention deficit hyperactivity disorder (ADHD) who are also smokers (*13*), adults with schizophrenia (*14*), women with severe obesity (*3*), or undergraduate students who screened positive for generalized anxiety disorder or social phobia (*8*).

### Sampling and measures

The median sample size was 117 participants, and the 25th percentile 47, and 75% percentile 170. One study had only 10 participants (*13*) and the largest sample had 4,318 participants (*4*).

### Schedule

Eighteen studies counted one wave of data collection, while the two others included six (*17, 18*). The monitoring period lasted between 2 and 60 days, with a median and mode of 7 days. Of the 20 included studies, two studies (*11, 12*) that collected data for four consecutive days did not report if monitoring days occurred during weekdays and/or week-end days.

### Technology used and training

The technologies used include mobile phones (*1, 3, 4, 7, 8, 9, 10, 12, 14, 15, 16, 18, 19, 20*), and computers (*5, 13*). Four studies did not mention what type of data collection modality was used. In nine of the studies, smartphones were supplied to the participants, two studies involved the use of participants' own phones, and in nine studies this information was not documented. Specific models of mobile phones were mentioned in some cases when devices were provided to participants (*2, 6, 7, 9, 11, 14, 19, 20*), such as Samsung Galaxy S4.2 (*9*), Samsung Galaxy S8 (*14*), Apple iPhone 5c (*2, 20*) and LG-P509 (*19*). The operating systems of the mobile phones varied, with Android and iOS being the most commonly mentioned. Different apps or programs were used for data collection, including home-developed apps such as the Motivation and Skills Support (MASS) (*14*), Happyhier (*4*), Ethicadata (*7*), EMA WebApps (*16*), Addressing People and Place Microenvironments (APP-Me) (*3*), and commercially available apps like Movisens (1) and Mood Triggers (*8*). The type of sensor used to capture geographic location varied across studies. In some cases, the EMA was conducted directly on the mobile phone itself with the location sourced from the smartphone location subsystem. Other studies used additional devices or apps such as GPS loggers (*9*), wearable devices like iBlue (*13*) or Garmin(e), and web-based EMA platforms (*12*).

Eight out of twenty studies reported providing training to participants, about how to operate the smartphone that was provided (*1, 9, 14, 19*), how to complete EMA surveys (*7, 9, 13, 14*), sometimes with mock EMA assessments (*19*), or practicing event-based triggered responses, as for each cigarette smoked (*13*). One study also provided information about how to interpret the EMA questions (*7*). Several studies provided specific instructions to help participants complete EMA surveys rapidly after being prompted (*6, 10*). Several studies also provided written instructions or a training guidebook to bring home (*9, 19*) or provided support options (*1*).

### Prompting strategy

Five studies used a fixed Interval Contingent strategy (e.g., prompts were set for certain times that were not random), and the number of prompts per day varied between 2 and once per hour during waking hours (*3, 8, 16, 16, 17*). The prompting schedule varied between studies: after school and in the evening on Thursday and Friday, and during the same time period on Saturday (4:00 p.m. and 9:00 p.m) (1), once per hour during waking hours (*8*), and at 08:00, 12:00, 16:00 and 20:00 (*16, 17, 18*). Using [60] taxonomy, five studies employed a Semi-random Interval Contingent strategy (EMAs were administered at random intervals within predetermined time windows) (*3, 7, 9, 14, 20*), with 2 to 12 prompts per day. Five studies used a Random Interval Contingent strategy (e.g., random times throughout each day) (*6, 10, 11, 12, 19*), with 3 to 6 prompts per day. Four studies used an Event-Based strategy (e.g., either through self-initiation of questionnaire at determined event, such as after being active for more than 30 min, prior to and following smoking, or through geofencing initiation, such as when the device detects being in a predetermined location, such as when entering a park.) (*1, 4, 5, 13*). One study did not report the strategy employed (*15*). Most studies did not report the maximum delay allowed between a prompt and filling the questionnaire. Three studies used a 30-minute delay limit (*10, 17, 18*), and one 20 minutes (*13*). Duration of prompt interval, meaning the duration between each prompt, was not reported in 11 studies (*1, 3, 5, 6, 8, 10, 11, 12, 14, 15, 19*) - as it does not apply to random interval contingent prompting strategy. One study reported five hours (*2*), two reported four hours (*16, 20*), three reported three to four hours (*9, 17, 18*), one reported a minimum of 90 minutes (*7*) and two studies using geofencing event-based strategy reported at least 50 minutes (*4*), and every 2.5 hours during waking hours (*13*).

### Measurement instruments

The instruments used in the reviewed studies varied across domains and variables of interest. Fifteen studies did not report any specific instrument used for measurement. However, for the assessment of social interactions and social functioning, two commonly employed instruments were the Heinrichs Quality of Life Scale — Interpersonal Relations subscale (QLS-IR) and the Birchwood Social Functioning Scale (SFS) (*14*). The measurement of affective states was done with the Positive Affect Negative Affect Schedule - Expanded Edition (PANAS-X) and the Multidimensional Experiential Avoidance Questionnaire (MEAQ)'s behavioral scale (*8*), which was modified to better align with the momentary assessment paradigm. In one study, the taxonomy of social activities developed by Levasseur et al. (2010) (*1*) served as a basis to assess social interactions, with specific questions co-designed and evaluated by users. Other instruments, such as the Profile of Mood States questionnaire, 2nd Edition–Youth (POMS-Y), were also used, although specific details were not provided in the reviewed studies (*5*). Interestingly, only one study used an instrument that was specifically designed and validated for an EMA context - that is in the context of repeated momentary measures (*1*).

### Main variables of interest

Momentary variables of interest related to health behaviors (diet, drinking, smoking, or smoking urge), mental health and well-being (mood, affective states, psychological stress or feelings of anxiety and momentary happiness), or health symptoms (physiological symptoms of distress, fatigue, and pain). One study included measures of perceived safety (*l*) and 5 studies also included measures of momentary social interactions (*1, 3, 7, 9, 11*). One common theme that emerges is the examination of adolescents' alcohol consumption and disorganization (*2*). Another area of investigation was the relationship between daily happiness (*2, 15, 16*) and various factors such as social motivation (*14*), social interaction (*1, 3, 7, 9, 11*), and contextual well-being (*1, 5, 6, 8, 9, 10, 11, 13*). These studies aimed to shed light on the determinants of individuals' happiness on a day-to-day basis (*4, 15, 16*), considering both internal and external factors that may influence one's emotional state.

Three studies (*12, 17, 20*) focused on momentary psychological stress. Multiple studies explored the triggers and consequences of momentary stress, including its association with mood, tiredness, and fatigue (*9, 11, 20*). Moreover, smoking urges (*13, 19*) were examined as a focal point in understanding addiction and behavioral patterns. Some studies also used measures of both physical and emotional well-being, with assessment of affect (*20*), pain, and fatigue levels (*10, 20*). The connection between lifestyle choices and emotional states is also explored, for example relating eating and drinking habits (*2, 3*) with happiness and psychological well-being. Lastly, substance use (*12*), anxiety and avoidance (*8*), and the concentration of nature on mood (*5*) were investigated as outcome variables in other studies.

### GPS recording

GPS coordinates were recorded at various frequencies. Eleven studies used continuous recording with varying intervals, six used momentary recording (at each EMA response), and three studies did not provide information about the recording intervals. The frequency used during continuous recordings varied across studies, with some sampled every 15 seconds (*5, 13*), while others were collected at 1-minute (*2, 17*), or 5-minute (*7, 9, 14*), or could be self-defined by the participants, at either 2, 5, 10, 30, or 60 minutes (*15*) intervals. Additionally, some studies recorded GPS data at each EMA prompt (*1, 6, 11, 12, 16, 19*). Moreover, one study implemented a unique approach where GPS recordings occurred at regular time intervals for participants with an Android OS smartphone, while participants with iOS smartphones had GPS position recorded only when they were changing location (*4*). The diversity of recording intervals used in these studies highlights the flexibility and adaptability of EMA in capturing real-time data in ecological contexts.

### Deriving environmental exposure from GPS locations

Several methods were used to derive environmental exposures and link these to EMA responses. Most studies used buffer calculations (*N*=9), which allowed authors to assess exposure to predefined factors within a certain distance, such as presence or number of alcohol, tobacco, or food outlets (*2, 6, 13, 19*) and physical environment variables (e.g., population density, type of land use, traffic noise, weather, or exposure to greenery) (*4, 9, 10, 16, 18*). The size of buffers ranged from 50m to 1,600m in the reviewed studies. Direct spatial overlay was the second most used technique (*N*=4). Using this method, researchers were able to characterize participants' locations in terms of their social and physical environments, by overlaying Census data to establish local social indicators (*11, 12, 15*), or Google Place API and weather datasets to identify the type of location and meteorological conditions (*8*). Two studies used prompted recall diaries to identify the type of locations the participants had visited during the day (*17, 18*), one of which completed location identification with spatial segmentation to distinguish between indoor and outdoor locations (*18*). Two other studies resorted to Google Maps to retrieve specific information about participants’ location, such as the type of location (*8*), or to identify if the participant was in a green space (*5*). One article used viewshed analysis to derive exposure to greenness (*1*). Some studies also used the location data to derive daily mobility metrics, whether significant locations detection, identifying the number of places where participants had stayed for at least 10 minutes (*13*); measuring convex hulls (*15*) or using trajectory imputation methods (*7*) to identify periods of movements and periods of pauses.

### Compliance and GPS match

Compliance, that is, the proportion of received prompts that were answered, was reported in 17 studies, and varied from 50% (*l*) to 100% (*d, k*), and three studies did not report compliance rate (*5, 7, 14*). No study reported if compliance varied by demographic or time-varying variables. Eight studies reported the proportion of EMA prompts for which a GPS coordinate was obtained: 46% (*19*), 56% (*12*), 75% (*9*), 76% (*4*), 86% (*8*), 99% (*11*), and 100% (*13, 16*). No study reported statistics on latency, that is, the delay between a prompt and an answer. No study reported how many prompts have been received.

### Modeling approaches

One study limited itself to descriptive statistics as the sole analytical approach without employing any other models (*13*). Modeling approaches include the use of zero-inflated Poisson models to handle excess of zeros in the data (*2*), multilevel models, and structural equation models (SEM) with cluster-robust estimation methods for handling clustering effects (*18*). More precisely, multilevel modeling (MLM) was the main approach used, with different configurations, including multilevel logistic regression (*3, 20*), multilevel linear models (*1, 4, 5, 9, 14, 15, 19*), and multilevel ordinal regression models (*10, 16, 17*). All the studies using multilevel approaches nested the data into two levels — answered prompts within individuals —, except for one study that used three levels, with assessments nested within days within individuals (*19*). Additionally, bivariate correlations (*7*), and generalized estimating equations (*12*) were applied. A study employed logistic regression models with individual fixed effects (*6*), and multi-layered personalized deep-learning models with temporal patterns (*8*).

## Guideline development

Consensus on the proposed guideline was reached after two rounds of interactions between the core team and each of the three experts consulted individually. Following the guideline development process, we ended up with a proposed STROBE-GEMA extension that includes a total of 27 categories (plus 4 subcategories), combining a total of 70 items. The 22 categories and 32 items from the original STROBE guideline have been integrated in our GEMA guideline. Eight categories and 6 items from the CREMAS guideline have been included to our guideline. We created one new category (namely “Consent”) and added 32 new items specific to GEMA studies. Below, Table 2 shows the guideline extension.

**Table 2:** STROBE-GEMA guideline

**Item**	**Item #**	**STROBE Recommendations**	**Item #**	**Extension for GEMA studies (STROBE-GEMA)**
**Title and abstract**				
** Title and abstract**	1	(a) Indicate the study’s design with a commonly used term in the title or the abstract (b) Provide in the abstract an informative and balanced summary of what was done and what was found	1	(c) Include geographic ecological momentary assessment in the abstract and keywords
** Background/** **rationale**	2	(a) Explain the scientific background and rationale for the investigation being reported	2	(b) Provide rationale for using GEMA for this study or topic of interest (e.g., to examine time and space variant/momentary environmental predictors (e.g., amount of greenness) of active daily mobility)
** Objectives**	3	State-specific objectives, including any prespecified hypotheses		
**Methods**				
** Study design**	4	Present key elements of study design early in the paper		
** Monitoring period**			4.1	State the number of days each wave of the study lasted, and how many weekdays versus weekend days^a^
** Prompting design**			4.2	(a) Describe prompting frequency (e.g. number of prompts per period of time) and associated rules (e.g., event based, geofencing trigger, schedule-based with random component, number of prompts per day, time available for completion) (b) Describe prompt notification type when not self-triggered (e.g. app notification, text message) (c) Describe prompting design for other variables of interest
** Training**			4.3	Indicate if, and how training of participants for GEMA protocol was conducted
** Consent**			4.4	a) Provide information about consent form and the ethical board certificate number b) Indicate location precision data collection as per local ethical committee ruling
** Setting**	5	Describe the setting, locations, and relevant dates, including periods of recruitment, exposure, follow-up, and data collection		
** Technology**			6	(a) Describe what technology was used, both for the momentary questionnaires (e.g. name of the application used for EMA prompts) and for capturing geographic location (e.g. mobile phone with or without separate GPS receiver, assisted GPS, laptop, wifi-based, self-report map-based question) and specify if the technology was provided to the participants or if they used their own device (e.g. cellphone) (b) Report any supplementary devices or embedded sensors used to measure other variables of interest (e.g. light, noise. physical activity) and provide relevant information regarding their use (c) Indicate if the technology used real-time or delayed data transmission (d) Indicate rules for technology use if any (e.g. GPS-tracker to be worn at the hip at all times, mobile phone to be carried at all times, etc.) (e) Indicate what app or web-app was used (if applicable) for data collection (f) Indicate if the technology used required recharging
** Participants**	7	**(a) *Cohort study***—Give the eligibility criteria, and the sources and methods of selection of participants. Describe methods of follow-up ***Case-control study***—Give the eligibility criteria, and the sources and methods of case ascertainment and control selection. Give the rationale for the choice of cases and controls ***Cross-sectional study***—Give the eligibility criteria, and the sources and methods of selection of participants **(b) *Cohort study***—For matched studies, give matching criteria and number of exposed and unexposed ***Case-control study***—For matched studies, give matching criteria and the number of controls per case		
** Variables**	8	(a) Clearly define all outcomes, exposures, predictors, potential confounders, and effect modifiers. Give diagnostic criteria, if applicable	8	(b) Provide information for variables derived from EMA questionnaires, and GPS or other location variables available that were used (Lat/Long; number of visible satellites, indoor/outdoor indicator) (c) Clearly define all environmental exposure variables (d) Provide temporal and spatial resolution of main EMA outcomes and covariates
** Data sources/ measurement**	9	(a) For each variable of interest, give sources of data and details of methods of assessment (measurement). Describe the comparability of assessment methods if there is more than one group	9	(b) Describe what geographic coordinates and metadata were available (e.g. Latitude/Longitude, number of visible satellites, HDOP/PDOP, indoor/outdoor indicator) (c) Describe how location coordinates were used to derive momentary or cumulative environmental exposure(s) (e.g. distance to exposure, density, use of buffers) (d) Describe temporal relation between EMA questions answered and location data (e.g. simultaneous punctual, cumulative location tracking during X minutes previous to answer to prompt) (e) Describe measurement instruments for all EMA questions, and if the instrument has been validated in an EMA context. (f) Describe possible distribution of instruments through prompts (i.e. what questions came with what prompt) (g) Specify the exact source(s) of location data (self-reported, device-based passive data collection, combination of both)
** Bias / Design features**	10	(a) Describe any efforts to address potential sources of bias	10	(b) Describe any design feature to address potential sources of bias (eg, reactivity) or participant burden (eg, EMA questions appearing in different orders) or spatial bias (including mitigation efforts to reduce potential selective daily mobility bias, the collection of widely-varied amounts of geo-data from individuals (e.g., those that move around more) and then running analyses that do not properly weight for such within and between person sources of geographically-specific sampling bias, accuracy of location variables)
** Study size**	11	Explain how the study size was arrived at	11	(a) Explain how the study size was arrived at, both in terms of number of participants and number of surveys
** Quantitative variables**	12	Explain how quantitative variables were handled in the analyses. If applicable, describe which groupings were chosen and why		
** Statistical methods (model)**	13	(a) Describe all statistical methods, including those used to control for confounding (b) Describe any methods used to examine subgroups and interactions (c) Explain how missing data were addressed (d) *Cohort study*—If applicable, explain how the loss to follow-up was addressed *Case-control study*—If applicable, explain how the matching of cases and controls was addressed *Cross-sectional study*—If applicable, describe analytical methods taking account of the sampling strategy (e) Describe any sensitivity analyses	13	(f) Indicate the number of levels of analysis (e.g., whether the statistical unit of analysis was the prompt, the day, or the individual) (g) Clearly explain and justify the number of levels and nesting structure used if multilevel analysis was used as often recommended (e.g. prompts within days within individuals)
**Results**				
** Participants / Attrition**	14	(a) Report numbers of individuals at each stage of study—e.g., numbers potentially eligible, examined for eligibility, confirmed eligible, included in the study, completing follow-up, and analyzed (b) Give reasons for non-participation at each stage (c) Consider the use of a flow diagram	14	(d) Define what is valid data and indicate the proportion of invalid data (e) Absence/Presence of GPS coordinate when momentary questionnaire is answered (f) Indicate participant attrition throughout the study; report attrition rates both by monitoring days and waves, if applicable^a^
** Prompt delivery and completion**^**a**^			15	(a) Report number of EMA prompts that were planned to be delivered. If possible, also report the number of EMA prompts that were actually received by participants and indicate reasons for why prompts were not sent out (eg, technical issues or participant noncompliance reason such as phone was powered off)^a^
** Latency**^**a**^			16	Report the amount of time from prompt signal to answering of prompt^a^
** Compliance rate**^**a**^			17	(a) Report total answered EMA prompts across all subjects and the average number of EMA prompts answered per person. Report compliance rate both by monitoring days, time of day and waves, if applicable. Indicate reasons for noncompliance, if known^a^ (b) Report proportion of participants who did not allow location data capture, if available
** Descriptive data **	18	(a) Give characteristics of study participants (e.g. demographic, clinical, social) and information on exposures and potential confounders (b) Indicate the number of participants with missing data for each variable of interest (c) *Cohort study*—Summarize follow-up time (e.g., average, and total amount)		
** Missing data**^**a**^			19	(a) Report whether EMA compliance is related to demographic or time-varying variables^a^ (b) Report proportion of answered prompts with and without location data (e.g. missing GPS coordinates), and whether missing GPS data is related to demographic, time-varying variables or hardware/OS used if available (e.g. smartphone model, OS version)
** Outcome data**	20	*Cohort study*—Report numbers of outcome events or summary measures over time *Case-control study—*Report numbers in each exposure category, or summary measures of exposure *Cross-sectional study—*Report numbers of outcome events or summary measures		
** Main results**	21	(a) Give unadjusted estimates and, if applicable, confounder-adjusted estimates and their precision (eg, 95% confidence interval). Make clear which confounders were adjusted for and why they were included (b) Report category boundaries when continuous variables were categorized (c) If relevant, consider translating estimates of relative risk into absolute risk for a meaningful time period		
** Other analyses**	22	Report other analyses done—eg analyses of subgroups and interactions, and sensitivity analyses		
**Discussion**				
** Key results**	23	Summarize key results with reference to study objectives		
** Limitations**	24	Discuss the limitations of the study, taking into account sources of potential bias or imprecision. Discuss both the direction and magnitude of any potential bias		(a) Discuss limitations of the study, taking into account sources of potential bias when using GEMA methods (e.g., reactivity, use of technology, sample selection bias due non answered EMA prompt or missing geographic location)
** Interpretation / Conclusions**	25	a) Give a cautious overall interpretation of results considering objectives, limitations, multiplicity of analyses, results from similar studies, and other relevant evidence		b) Discuss whether and how GEMA was relevant to address the research question
** Generalizability**	26	Discuss the generalizability (external validity) of the study results		
**Other information**				
** Funding**	27	Give the source of funding and the role of the funders for the present study and, if applicable, for the original study on which the present article is based		

^a^ Adopted from CREMAS checklist

## Discussion and conclusion

The aims of this study were twofold: 1) Conduct a systematic review of GEMA studies that use sensor-based location data to construct and relate measures of environmental exposures and/or experiences with behavioral or health outcomes; and 2) Develop a STROBE extension guideline for GEMA studies. An important strength of this guideline is that it has been refined in collaboration with international GEMA experts.

As happens with some projects, our initial goal was only limited to the first part, but the review showed us important gaps and inconsistencies in how GEMA studies are currently reported, leading us to extend our work with this second objective of guideline development. The review identified a total of 20 studies that met our inclusion criteria. These came from a wide range of disciplines, and were all published in distinct journals, underlining the variety of fields in which this method is increasingly being used.

Heterogenous reporting conventions highlight the need for standards to unify reporting elements and more efficiently develop a useful actionable knowledge base for future research. Based on this review’s results, and on existing STROBE and CREMAS guidelines, a STROBE-GEMA guideline was developed to propose recommendations to report future GEMA studies. The guideline has a total of 27 categories. A total of 20 additional items, in 10 of 22 of the original STROBE categories, 12 other additional items, in 6 of the 16 of the original CREMAS categories, and 1 item in 1 new category (consent) were added. While this review aimed at identifying a systematic way to report GEMA studies, this guideline does not serve as a GEMA study design recommendation. However, it can give the researchers an overview of what factors are important to consider when designing a GEMA study.

When creating this guideline, several key concerns were raised regarding GPS data collection and treatment. While there are agreed upon minimal wearing time thresholds for accelerometry data when aiming to measure daily physical activity [6], there is no such equivalent to evaluate how much missing location data can affect - or not - a GEMA study, although there are ways to ensure that the amount of time under observation (aka, the denominator) is balanced across participants, which can alleviate some uncertainty.

Missing location data may sometimes truly be linked to participants’ refusal to share their location, but it is most often due nothing to other compliance factors. Spending time in locations that are not reached by GPS signals, such as underground transportation systems, and many other indoor places, are challenges related to the technology itself. Interesting methodological developments in geographic imputation can provide location estimates of missing activity space data in GEMA studies. The Socio-spatial Adolescent Study, conducted in Richmond, Virginia, collected data among 247 adolescents between 2012 and 2014. Using relatively simple geographic imputation techniques, either imputing (artificially removed) missing data through a random selection among the known locations (Census tracts) for a given individual or imputing missing data with a person’s activity space centroid location showed good model performance [40]. Also complicating matters is the fact that geolocation accuracy (how close the measure is to what it should be measuring) and precision (how clustered repeated measures are), when location coordinates are available, can often be difficult to assess, because the ‘true’ location is most often unknown. In that same GEMA study with adolescents held in Richmond, among 3718 GEMA answers from 72 participants who reported being at home and for which precise residential home location was available, 76% of GPS points fell within half a mile, 61% within a quarter mile, and 48% within a sixteenth of a mile of participants actual home location [39]. Some devices may provide complete GPS NMEA sentences that include metrics such as Dilution of Precision or number of visible satellites, which can help estimate spatial accuracy, but most often, these complete sentences are not available.

When we do have a-priori hypotheses about place effects requiring within-day, perhaps time-of-day level precision, a key consideration is whether the research content area focus is more on place-based exposures (e.g., inhaling particulate matter) versus subjective experiences (e.g., experiencing acute distress). This also leads to an important distinction between objective (GIS) and subjective (self-reported) environmental measures. Location can today be tracked passively - that is, without any active input from the participant beyond his/her consent and her willingness to carry a smartphone or any other type of device that contains a GPS receiver [24]. However, additional information such as the type of activities being conducted, specific health behaviors such as diet or tobacco consumption, current emotional states and social interactions still require active self-report. This data collection is often conducted using electronic diaries or short questionnaires.

While we believe the detailed collection of within-day information about activity locations that GEMA can provide, especially when continuous GPS tracking is done, helps address the Uncertain Geographic Context Problem [31, 34], issues of causality remain to be better addressed. Indeed, the selective daily mobility bias, linked to the fact that people purposefully choose some destinations because of personal preference rather than accessibility or exposure makes it more difficult to assess directionality in observed momentary spatial exposures and correlated behavioral “outcomes” [5]. Additionally, while there is a large body of work on spatial distribution of both outcomes (e.g., psychological outcomes, health behaviors) and environmental exposures (e.g., ‘neighborhood research’), with inputs from various disciplines including geography, transportation, or environmental psychology, within-day and within-person variations in such people-place interactions have historically only recently started to be explored.

Conceptualizing place-effects as experiences lends naturally to what Cummins et al., [13] refer to as a “relational,” i.e., network-based, disaggregated, place-effects, which itself diverges in a number of interesting and potentially consequential ways from traditional cumulative exposure conceptions of place [65]. We can only assume that many essential psychometric developments remain yet unrealized within this still novel area of health research. The present guideline aims to establish reporting guidelines that are technically inclusive and comprehensive, and also future-oriented toward the high degree of uncertainty we face as these technologies continue to rapidly advance.

This guideline can be used with consideration of other sensor-measured environmental and behavioral factors such as light, noise, physical activity. Since reporting guidelines for such variables was out of the scope of our work, existing guidelines can be used to correctly report these various dimensions. For example, [44] have published recommendations for reporting accelerometer measured physical activity intervention studies. There is also a promising potential to combine GEMA studies with qualitative mapping procedures [37, 38].

In conclusion, this is the first study with the initial aim to systematically review GEMA studies using sensor-based location data and related environmental exposures. Because of substantial reporting inconsistencies, we developed this STROBE-GEMA guideline. We believe this guideline will be useful to the large variety of GEMA studies that explore how the environments we live in influence subjective states, behaviors, and physiological parameters. As the variety and use of sensors for momentary assessments will increase, it might be combined with other sensor-specific reporting guidelines.

### Supplementary Information

Additional file 1. Data extraction. Description of data: Table of all extracted variables from the systematic review, for each included study.

## Data Availability

No datasets were generated or analysed during the current study.

## References

[1] Bader MD, Mooney SJ, Rundle AG. Protecting personally identifiable information when using online geographic tools for public health research. Am J Public Health. 2016;106(2):206–8. 10.2105/AJPH.2015.302951. 10.2105/AJPH.2015.302951 PMC4815822 26794375

[2] Bollenbach L, Schmitz J, Niermann C, Kanning M. How do people feel while walking in the city? Using walking-triggered e-diaries to investigate the association of social interaction and environmental greenness during everyday life walking. Front Psychol. 2022;13:970336. 10.3389/fpsyg.2022.970336 PMC9549356 36225697

[3] Byrnes HF, Miller BA, Morrison CN, Wiebe DJ, Woychik M, Wiehe SE. Association of environmental indicators with teen alcohol use and problem behavior: Teens’ observations vs. objectively-measured indicators. Health Place. 2017;43:151–7. 10.1016/j.healthplace.2016.12.004 PMC5285270 28061392

[4] Chaix B (2009). Geographic life environments and coronary heart disease: a literature review, theoretical contributions, methodological updates, and a research agenda. Annu Rev Public Health.

[5] Chaix B, Méline J, Duncan S, Jardinier L, Perchoux C, Vallée J, et al. Neighborhood environments, mobility, and health: Towards a new generation of studies in environmental health research. Revue d’Épidémiologie et de Santé Publique. 2013;61:S139–45. 10.1016/j.respe.2013.05.017 23845204

[6] Choi L, Liu Z, Matthews CE, Buchowski MS (2011). Validation of Accelerometer Wear and Nonwear Time Classification Algorithm. Med Sci Sports Exerc..

[7] Christensen TC, Barrett LF, Bliss-Moreau E, Lebo K, Kaschub C (2003). A practical guide to experience sampling procedures. J Happiiness Stud.

[8] Clark DO, Keith NR, Ofner S, Hackett J, Li R, Agarwal N (2022). Environments and situations as correlates of eating and drinking among women living with obesity and urban poverty. Obes Sci Pract..

[9] Colombo D, Fernández-Álvarez J, Patané A, Semonella M, Kwiatkowska M, García-Palacios A (2019). Current State and Future Directions of Technology-Based Ecological Momentary Assessment and Intervention for Major Depressive Disorder: A Systematic Review. J Clin Med..

[10] Conner TS, Mehl MR. Ambulatory Assessment: Methods for Studying Everyday Life. In: Emerging Trends in the Social and Behavioral Sciences. Hoboken: John Wiley & Sons, Ltd; 2015. p. 1–15. [cité 29 nov 2023]. Disponible sur: https://onlinelibrary.wiley.com/doi/abs/10.1002/9781118900772.etrds0010.

[11] Crouse DL, Pinault L, Christidis T, Lavigne E, Thomson EM, Villeneuve PJ (2021). Residential greenness and indicators of stress and mental well-being in a Canadian national-level survey. Environ Res.

[12] Cui Y, Eccles KM, Kwok RK, Joubert BR, Messier KP, Balshaw DM (2022). Integrating Multiscale Geospatial Environmental Data into Large Population Health Studies: Challenges and Opportunities. Toxics..

[13] Cummins S (2007). Commentary: Investigating neighbourhood effects on health—avoiding the ‘Local Trap’. Int J Epidemiol..

[14] Cuschieri S (2019). The STROBE guidelines. Saudi J Anaesth..

[15] Dao KP, Cocker KD, Tong HL, Kocaballi AB, Chow C, Laranjo L. Smartphone-Delivered Ecological Momentary Interventions Based on Ecological Momentary Assessments to Promote Health Behaviors: Systematic Review and Adapted Checklist for Reporting Ecological Momentary Assessment and Intervention Studies. JMIR Mhealth Uhealth. 2021;9(11):e22890. 10.2196/22890. 10.2196/22890 PMC8663593 34806995

[16] Delespaul P, deVries M, van Os J (2002). Determinants of occurrence and recovery from hallucinations in daily life. Soc Psychiatry Psychiatr Epidemiol..

[17] de Vries S, Nieuwenhuizen W, Farjon H, van Hinsberg A, Dirkx J. In which natural environments are people happiest? Large-scale experience sampling in the Netherlands. Landsc Urban Plann. 2021;205:103972.

[18] Dora J, Piccirillo M, Foster KT, Arbeau K, Armeli S, Auriacombe M (2023). The daily association between affect and alcohol use: A meta-analysis of individual participant data. Psychol Bull..

[19] Elliston KG, Schüz B, Albion T, Ferguson SG. Comparison of Geographic Information System and Subjective Assessments of Momentary Food Environments as Predictors of Food Intake: An Ecological Momentary Assessment Study. JMIR Mhealth Uhealth. 2020;8(7):e15948. 10.2196/15948 PMC7407250 32706728

[20] Ettema D, Smajic I (2015). Walking, places and wellbeing. Geograph J..

[21] Fresán U, Bernard P, Fabregues S, Boronat A, Araújo-Soares V, König LM, et al. A Smartphone Intervention to Promote a Sustainable Healthy Diet: Protocol for a Pilot Study. JMIR Res Protoc. 2023;12(1):e41443. 10.2196/41443. 10.2196/41443 PMC10020902 36862497

[22] Fulford D, Mote J, Gonzalez R, Abplanalp S, Zhang Y, Luckenbaugh J (2021). Smartphone sensing of social interactions in people with and without schizophrenia. J Psychiatr Res..

[23] Fuller D, Sharek M, Stanley K (2017). Ethical implications of location and accelerometer measurement in health research studies with mobile sensing devices. Soc Sci Med.

[24] Gharani P, Karimi HA, Syzdykbayev M, Burke LE, Rathbun SL, Davis EM (2021). Geographically-explicit Ecological Momentary Assessment (GEMA) Architecture and Components: Lessons Learned from PMOMS. Inform Health Soc Care..

[25] Heron KE, Smyth JM (2010). Ecological momentary interventions: Incorporating mobile technology into psychosocial and health behaviour treatments. Brit J Health Psychol..

[26] Higdon D, Swall J, Kern J. Non-Stationary Spatial Modeling. arXiv. 2022. [cité 12 sept 2023]. Disponible sur: http://arxiv.org/abs/2212.08043.

[27] Jacobson NC, Bhattacharya S. Digital biomarkers of anxiety disorder symptom changes: Personalized deep learning models using smartphone sensors accurately predict anxiety symptoms from ecological momentary assessments. Behav Res Ther. 2022;149:104013. 10.1016/j.brat.2021.104013 PMC8858490 35030442

[28] Kamalyan L, Yang JA, Pope CN, Paolillo EW, Campbell LM, Tang B (2021). Increased Social Interactions Reduce the Association Between Constricted Life-Space and Lower Daily Happiness in Older Adults With and Without HIV: A GPS and Ecological Momentary Assessment Study. Am J Geriatr Psychiat..

[29] Kerr J, Duncan S, Schipperjin J (2011). Using Global Positioning Systems in Health Research: A Practical Approach to Data Collection and Processing. Am J Prevent Med..

[30] Kirchner TR, Cantrell J, Anesetti-Rothermel A, Ganz O, Vallone DM, Abrams DB (2013). Geospatial Exposure to Point-of-Sale Tobacco: Real-Time Craving and Smoking-Cessation Outcomes. Am J Prevent Med..

[31] Kirchner TR, Gao H, Lewis DJ, Anesetti-Rothermel A, Carlos HA, House B (2019). Individual Mobility and Uncertain Geographic Context: Real-time Versus Neighborhood Approximated Exposure to Retail Tobacco Outlets Across the US. J Healthc Inform Res..

[32] Kirchner TR, Shiffman S (2016). Spatio-temporal determinants of mental health and well-being: advances in geographically-explicit ecological momentary assessment (GEMA). Soc Psychiat Psychiatr Epidemiol..

[33] Kondo MC, Triguero-Mas M, Donaire-Gonzalez D, Seto E, Valentín A, Hurst G, et al. Momentary mood response to natural outdoor environments in four European cities. Environ Int. 2020;134:105237. 10.1016/j.envint.2019.105237 31677802

[34] Kwan MP (2012). The Uncertain Geographic Context Problem. Ann Assoc Am Geograph..

[35] Li D, Deal B, Zhou X, Slavenas M, Sullivan WC (2018). Moving beyond the neighborhood: Daily exposure to nature and adolescents’ mood. Landscape Urban Plann..

[36] Liao Y, Skelton K, Dunton G, Bruening M. A Systematic Review of Methods and Procedures Used in Ecological Momentary Assessments of Diet and Physical Activity Research in Youth: An Adapted STROBE Checklist for Reporting EMA Studies (CREMAS). J Med Internet Res. 2016;18(6):e151. 10.2196/jmir.4954. 10.2196/jmir.4954 PMC4933800 27328833

[37] McQuoid J, Thrul J, Ling P (2018). A geographically explicit ecological momentary assessment (GEMA) mixed method for understanding substance use. Soc Sci Med.

[38] McQuoid J, Thrul J, Lopez-Paguyo K, Ling PM. Exploring multiple drug use by integrating mobile health and qualitative mapping methods - An individual case study. Int J Drug Policy. 2021;97:103325. 10.1016/j.drugpo.2021.103325. 10.1016/j.drugpo.2021.103325 PMC8585680 34175527

[39] Mennis J, Mason M, Ambrus A, Way T, Henry K (2017). The spatial accuracy of geographic ecological momentary assessment (GEMA): Error and bias due to subject and environmental characteristics. Drug Alcohol Dependence.

[40] Mennis J, Mason M, Coffman DL, Henry K (2018). Geographic Imputation of Missing Activity Space Data from Ecological Momentary Assessment (EMA) GPS Positions. Int J Environ Res Public Health..

[41] Mennis J, Mason M, Light J, Rusby J, Westling E, Way T (2016). Does substance use moderate the association of neighborhood disadvantage with perceived stress and safety in the activity spaces of urban youth?. Drug Alcohol Depend..

[42] Mitchell JT, Schick RS, Hallyburton M, Dennis MF, Kollins SH, Beckham JC (2014). Combined ecological momentary assessment and global positioning system tracking to assess smoking behavior: a proof of concept study. J Dual Diagn..

[43] Moher D, Schulz KF, Simera I, Altman DG. Guidance for Developers of Health Research Reporting Guidelines. PLOS Med. 2010;7(2):e1000217. 10.1371/journal.pmed.1000217 PMC2821895 20169112

[44] Montoye AHK, Moore RW, Bowles HR, Korycinski R, Pfeiffer KA (2018). Reporting accelerometer methods in physical activity intervention studies: a systematic review and recommendations for authors. Br J Sports Med..

[45] Mow JL, Gard DE, Mueser KT, Mote J, Gill K, Leung L (2022). Smartphone-based mobility metrics capture daily social motivation and behavior in schizophrenia. Schizophr Res..

[46] Palmer JRB, Espenshade TJ, Bartumeus F, Chung CY, Ozgencil NE, Li K. New Approaches to Human Mobility: Using Mobile Phones for Demographic Research. 2013;24. 10.1007/s13524-012-0175-z PMC3633623 23192393

[47] Perski O, Kwasnicka D, Kale D, Schneider V, Szinay D, ten Hoor G (2023). Within-person associations between psychological and contextual factors and lapse incidence in smokers attempting to quit: A systematic review and meta-analysis of ecological momentary assessment studies. Addiction..

[48] Reichert M, Giurgiu M, Koch ED, Wieland LM, Lautenbach S, Neubauer AB, et al. Ambulatory assessment for physical activity research: State of the science, best practices and future directions. Psychol Sport Exerc. 2020;50:101742. 10.1016/j.psychsport.2020.101742. 10.1016/j.psychsport.2020.101742 PMC7430559 32831643

[49] Rojas-Rueda D, Nieuwenhuijsen MJ, Gascon M, Perez-Leon D, Mudu P (2019). Green spaces and mortality: a systematic review and meta-analysis of cohort studies. Lancet Planet Health..

[50] Senanayake N, King B (2021). Geographies of uncertainty. Geoforum.

[51] Smith KE, Juarascio A (2019). From Ecological Momentary Assessment (EMA) to Ecological Momentary Intervention (EMI): Past and Future Directions for Ambulatory Assessment and Interventions in Eating Disorders. Curr Psychiatry Rep..

[52] Stone AA, Shiffman S (1994). Ecological Momentary Assessment (Ema) in Behavioral Medicine. Ann Behav Med..

[53] Su L, Zhou S, Kwan MP, Chai Y, Zhang X (2022). The impact of immediate urban environments on people’s momentary happiness. Urban Stud..

[54] Tao Y, Chai Y, Kou L, Kwan MP. Understanding noise exposure, noise annoyance, and psychological stress: Incorporating individual mobility and the temporality of the exposure-effect relationship. Appl Geogr. 2020;125:102283.

[55] Tao Y, Kou L, Chai Y, Kwan MP. Associations of co-exposures to air pollution and noise with psychological stress in space and time: A case study in Beijing, China. Environ Res. 2021;196:110399. 10.1016/j.envres.2020.110399 33157109

[56] Trull TJ, Ebner-Priemer UW (2020). Ambulatory Assessment in Psychopathology Research: A Review of Recommended Reporting Guidelines and Current Practices. J Abnorm Psychol.

[57] Twohig-Bennett C, Jones A (2018). The health benefits of the great outdoors: A systematic review and meta-analysis of greenspace exposure and health outcomes. Environ Res.

[58] Watkins KL, Regan SD, Nguyen N, Businelle MS, Kendzor DE, Lam C, et al. Advancing Cessation Research by Integrating EMA and Geospatial Methodologies: Associations Between Tobacco Retail Outlets and Real-time Smoking Urges During a Quit Attempt. Nicotine Tob Res. 2014;16(Suppl 2):S93–101. 10.1093/ntr/ntt135. 10.1093/ntr/ntt135 PMC3977633 24057995

[59] World Health Organization. Ottawa Charter for Health Promotion, 1986. Regional Office for Europe. 1986;6. https://iris.who.int/handle/10665/349652.

[60] Wrzus C, Mehl MR (2015). Lab And/Or Field? Measuring Personality Processes and Their Social Consequences. Eur J Pers.

[61] York Cornwell E, Goldman AW (2020). Neighborhood Disorder and Distress in Real Time: Evidence from a Smartphone-Based Study of Older Adults. J Health Soc Behav..

[62] Yu R, Cheung O, Lau K, Woo J (2017). Associations between Perceived Neighborhood Walkability and Walking Time, Wellbeing, and Loneliness in Community-Dwelling Older Chinese People in Hong Kong. Int J Environ Res Public Health..

[63] Zamanifard H, Alizadeh T, Bosman C, Coiacetto E (2019). Measuring experiential qualities of urban public spaces: users’ perspective. J Urban Design..

[64] Zandbergen PA, Barbeau SJ (2011). Positional Accuracy of Assisted GPS Data from High-Sensitivity GPS-enabled Mobile Phones. J Nav..

[65] Zhang L, Zhou S, Kwan MP. The temporality of geographic contexts: Individual environmental exposure has time-related effects on mood. Health Place. 2023;79:102953. 10.1016/j.healthplace.2022.102953. 10.1016/j.healthplace.2022.102953 36512953

[66] Zhang X, Zhou S, Kwan MP, Su L, Lu J (2020). Geographic Ecological Momentary Assessment (GEMA) of environmental noise annoyance: the influence of activity context and the daily acoustic environment. Int J Health Geogr..

